# Supplementary material for: Ciliary Ultrastructure Assessed by Transmission Electron Microscopy in Adults with Bronchiectasis and Suspected Primary Ciliary Dyskinesia but Inconclusive Genotype
Source: Cells. 2023 Nov 18;12(22):2651. doi: 10.3390/cells12222651 (PMC10670349; doi:10.3390/cells12222651)
Supplement: Supplementary file 1 [file cells-12-02651-s001.zip › cells-2691768-supplementary.pdf]

Ciliary Ultrastructure Assessed by Transmission Electron Microscopy in Adults with Bronchiectasis and Suspected Primary Ciliary Dyskinesia but Inconclusive Genotype

Ben Ole Staar, Jan Hegermann, Bernd Auber, Raphael Ewen, Sandra von Hardenberg, Ruth Olmer, Isabell Pink, Jessica Rademacher, Martin Wetzke, and Felix C. Ringshausen

– Supplementary material –

**Supplementary Table S1.** Genes included in PCD screening panel
ARMC4, CCDC103, CCDC114, CCDC151, CCDC39, CCDC40, CCDC65, CCNO, CFTR, DNAAF1, DNAAF2, DNAAF3, DNAAF4, DNAAF5, DNAH1, DNAH11, DNAH3, DNAH5, DNAH8, DNAI1, DNAI2, DNAJB13, DNAL1, DRC1, GAS8, HYDIN, LRRC6, MCIDAS, NME8, OFD1, PIH1D3, RPGR, RSPH1, RSPH3, RSPH4A, RSPH9, SPAG1, ZMYND10
